# The efficacy of tolvaptan for heart failure in chronic kidney disease: A protocol for systematic review and meta-analysis

**DOI:** 10.1097/MD.0000000000032366

**Published:** 2022-12-30

**Authors:** Zhi-Yong Zhu, Meng Cui, Jie Zhao, Hong-Yun Wang

**Affiliations:** a Department of Nephrology, Zibo Central Hospital, Shandong, China.

**Keywords:** chronic kidney disease, heart failure, meta-analysis, protocol, tolvaptan

## Abstract

Approximately half of people with heart failure have chronic kidney disease (CKD). Tolvaptan is reported to be effective in treating heart failure. However, the safety and efficacy of its use in patients with CKD is uncertain. In this study, we conducted a protocol for systematic review and meta-analysis to investigate the efficacy and safety of tolvaptan on patients with heart failure and CKD.

**Methods:**

This study protocol has been registered in the PROSPERO and the registration number is CRD42022368148. The consent of this protocol report is based on the Preferred Reporting Items for Systematic Review and Meta-Analysis Protocols (PRISMA-P) 2015 statement guidelines. We will include randomized controlled trials related to tolvaptan in patients with heart failure and CKD. Two research members will electronically and independently search 4 English databases (EMBASE, PubMed, National Guideline Clearinghouse, and Cochrane Central Register of Controlled Trials) and 4 Chinese databases (Chinese Biomedical Literature Database, Chinese National Knowledge Infrastructure, Wanfang Database, and VIP Database) from their inception to November 2022. The risk of bias in each included study will be assessed utilizing the Cochrane Collaboration’s risk of bias tool. All statistical analyses will be conducted using the software program Review Manager version 5.3.

**Results:**

The results of this systematic review will be published in a peer-reviewed journal.

**Conclusion:**

This review can provide convincing evidence to help clinicians make decisions when dealing with heart failure and CKD.

## 1. Introduction

Patients with chronic kidney disease (CKD) have a greater risk of end-stage kidney disease and adverse cardiac events than patients with normal kidney function.^[1,2]^ CKD patients have an increased risk for structure-related heart disease, especially heart failure and arrhythmia.^[3,4]^ The majority of them will die of cardiovascular disease before developing end-stage renal disease. Therefore, improving cardiovascular function may improve the prognosis of patients with CKD.

The prevalence of heart failure and concomitant CKD is increasing.^[5,6]^ Additionally, CKD may increase the risk of harms associated with heart failure medication. While CKD accounts for a growing burden of morbidity and mortality in patients with HF, the management of this condition remains difficult.^[7]^ Generally, there is less use of heart failure medication as patients with heart failure and CKD may be more susceptible to the renal and metabolic effects of various heart failure therapies.^[5]^ Therefore, a better understanding of appropriate pharmacological management of heart failure in this patient population is particularly important.

Tolvaptan is a selective vasopressin V2 receptor antagonist that disturbs the movement of aquaporin 2 to the luminal side of cortical collecting duct cells by activating cyclic adenosine monophosphate.^[8,9]^ In addition, tolvaptan inhibits the reabsorption of water and produces water diuresis through a relatively recently identified mechanism of action.^[10]^ Tolvaptan is an alternative to the use of loop diuretics that is expected to slow the progression of renal failure and improve the prognosis of heart failure patients. Specifically, tolvaptan exerts a protective effect on the kidney by initiating a diuretic effect without activating the renin-angiotensin system. Additionally, it has been shown that renal blood flow and the glomerular filtration rate are not reduced by tolvaptan.^[11]^ In this study, we conducted a protocol for systematic review and meta-analysis to investigate the efficacy and safety of tolvaptan on patients with heart failure and CKD.

## 2. Methods

### 2.1. Registration

This study protocol has been registered in the PROSPERO and the registration number is CRD42022368148. The consent of this protocol report is based on the Preferred Reporting Items for Systematic Review and Meta-Analysis Protocols (PRISMA-P) 2015 statement guidelines.^[12]^

### 2.2. Inclusion criteria for study selection

#### 2.2..1. Study design.

We will include randomized controlled trials related to tolvaptan in patients with heart failure and CKD. Due to language restrictions, we will search for articles in English and Chinese.

#### 2.2..2. Participants.

We will include heart failure patients with CKD, regardless of sex, age, racial group, education, and economic status.

#### 2.2..3. Interventions and control.

Intervention group receives intravenous tolvaptan and control group receives any type of loop diuretics, regardless of dose and course.

#### 2.2..4. Outcomes measures.

Primary endpoints are estimated glomerular filtration rate and cardiac output. Secondary endpoints are the actual or percent change of serum creatinine, sodium, and brain natriuretic peptide within 72 hours after treatment.

### 2.3. Database search strategy

Two research members will electronically and independently search 4 English databases (EMBASE, PubMed, National Guideline Clearinghouse, and Cochrane Central Register of Controlled Trials) and 4 Chinese databases (Chinese Biomedical Literature Database, Chinese National Knowledge Infrastructure, Wanfang Database, and VIP Database) from their inception to November 2022. The searched items will be used as follows: tolvaptan, heart failure, CKD, and randomized controlled trial. The same terms will be searched in the Chinese databases. The data will be retrieved with the combination of medical keywords and uncontrolled terms. The detailed retrieval strategy of PubMed database will be shown in Table 1, and will be constantly modified by searching other databases.

**Table 1:** Search strategy for PubMed.

#1 heart failure [Title/Abstract]
#2 heart decompensation [Title/Abstract]
#3 cardiac failure [Title/Abstract]
#4 myocardial failure [Title/Abstract]
#5 #1 OR #2 OR #3 OR #4
#6 chronic kidney disease [Title/Abstract]
#7 kidney failure [Title/Abstract]
#8 renal failure [Title/Abstract]
#9 renal insufficiency [Title/Abstract]
#10 chronic renal disease [Title/Abstract]
#11 end-stage kidney [Title/Abstract]
#12 #6 OR #7 OR #8 OR #9 OR #10 OR #11
#13 tolvaptan [Title/Abstract]
#14 vasopressin V2 receptor antagonist [Title/Abstract]
#15 #13 OR #14
#16 #5 AND #12 AND #15

### 2.4. Study selection

**Figure 1.**
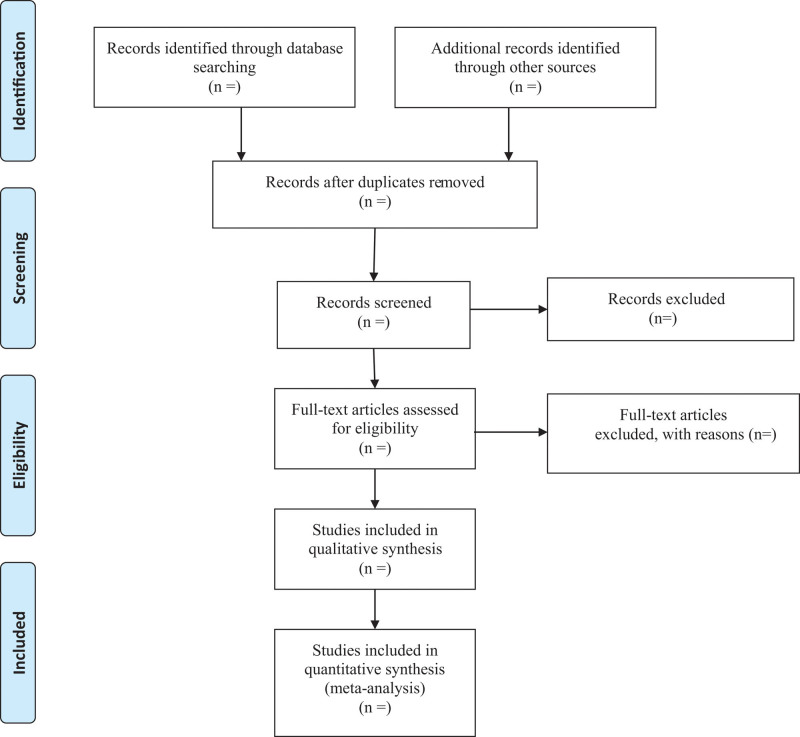
Flow diagram of study selection.

First of all, all qualified documents will be extracted in the form of title and abstract, and preliminary screening will be conducted based on this information. On the basis of the previous step, the full text of the qualified literature will be obtained and further screened. All screening processes will be performed independently by the 2 authors, and the reasons for each rejection will be documented. Disagreements between the 2 reviewers will be resolved by discussing with the third investigator. A PRISMA-compliant flow chart (Fig. 1) will be used to describe the selection process of eligible literatures.

### 2.5. Data extraction

The following data are extracted for each article: bibliographical data, including authors and year of publication; clinical trial features such as sample size, study flow, recruitment method, criteria for inclusion and exclusion, primary measures, time and point of assessments, and duration of the intervention; participant characteristics such as age, sex, and so on; patient background, including country and race; and study drop-out rate and handling of missing data.

### 2.6. Risk of bias assessment

Two investigators will separately assess the risk of bias of the included studies using the Cochrane risk of bias assessment tool.^[13]^ The evaluation of each study mainly included the following 7 aspects: random sequence generation, allocation hiding, blinding of participants and personnel, blinding of outcome assessment, incomplete outcome data, incomplete outcome data, selective outcome reporting, and other biases. Finally, the bias of the study will be rated on 3 levels: “low,” “high,” and “ambiguous.” Discrepancies will be addressed by consulting a third reviewer.

### 2.7. Statistical analysis

Differences between the intervention and control groups will be assessed. Mean differences (MDs) with 95% confidence intervals (CIs) will be used to measure the effects of treatment for continuous data. We will convert other forms of data into MDs. For outcome variables on different scales, we will use standard MDs with 95% CIs. For dichotomous data, we will present the treatment effects as relative risks with 95% CIs, and other binary data will be converted into relative risk values. All statistical analyses will be conducted using the software program Review Manager version 5.3 (Copenhagen, The Nordic Cochrane Centre, the Cochrane Collaboration) for Windows. We will contact the corresponding authors of the studies with missing information to acquire and verify the data, whenever possible. When appropriate, we will pool the data across studies to conduct a meta-analysis using fixed or random effects. Subgroup analyses will be performed to identify the possible causes in cases of heterogeneity (defined by results of tests of heterogeneity that indicate *P* < .1 via Chi-squared tests and Higgins *I*^2 ^≥ 50%). Sensitivity analysis will be also applied to evaluate the robustness and reliability of the combined results of included studies. Methodological quality, heterogeneity, studies quality and sample characteristic will be considered.

### 2.8. Ethics

Given that the meta-analysis is a secondary research which based on some previously published data, ethical approval is not necessary.

## 3. Discussion

Individuals with CKD are at high risk of cardiovascular morbidity and death.^[14,15]^ A narrative review including 1076,104 patients found a significantly higher risk of death among heart failure patients with comorbid CKD, compared to those without baseline CKD.^[16]^ They may have the potential for significant benefit more from interventions for heart failure, but they may also be more vulnerable to the potential harms of these interventions.^[17,18]^ In accordance with current guidelines, pharmacological interventions for heart failure should be used in caution in patients with comorbid renal insufficiency. This is the first meta-analysis to investigate the efficacy and safety of tolvaptan on patients with heart failure and CKD. Future research should focus on analyzing existing data of general population with heart failure to explore the effect in sub-groups of patients with CKD in order to yield valuable insights for the management of people with heart failure and CKD.

## Author contributions

**Conceptualization:** Meng Cui.

Investigation: Jie Zhao.

Writing – original draft: Zhi-Yong Zhu.

Writing – review & editing: Hong-Yun Wang.
